# Sex Differences in the Association between Serum Levels of Testosterone and Frailty in an Elderly Population: The Toledo Study for Healthy Aging

**DOI:** 10.1371/journal.pone.0032401

**Published:** 2012-03-05

**Authors:** Laure Carcaillon, Carmen Blanco, Cristina Alonso-Bouzón, Ana Alfaro-Acha, Francisco-José Garcia-García, Leocadio Rodriguez-Mañas

**Affiliations:** 1 Fundación para la Investigación Biomédica, Hospital Universitario de Getafe, Madrid, Spain; 2 Servicio de Análisis Clinicos, Hospital Universitario de Getafe, Madrid, Spain; 3 Servicio de Geriatría, Hospital Universitario de Getafe, Madrid, Spain; 4 Servicio de Geriatría, Hospital Virgen del Valle, Complejo Hospitalario de Toledo, Toledo, Spain; University of Valencia, Spain

## Abstract

**Background:**

Age-associated decline in testosterone levels represent one of the potential mechanisms involved in the development of frailty. Although this association has been widely reported in older men, very few data are available in women. We studied the association between testosterone and frailty in women and assessed sex differences in this relationship.

**Methods:**

We used cross-sectional data from the Toledo Study for Healthy Aging, a population-based cohort study of Spanish elderly. Frailty was defined according to Fried's approach. Multivariate odds-ratios (OR) and 95% confidence intervals (CI) associated with total (TT) and free testosterone (FT) levels were estimated using polytomous logistic regression.

**Results:**

In women, there was a U-shaped relationship between FT levels and frailty (p for FT^2^ = 0.03). In addition, very low levels of FT were observed in women with ≥4 frailty criteria (age-adjusted geometric means = 0.13 versus 0.37 in subjects with <4 components, p = 0.010). The association of FT with frailty appeared confined to obese women (p-value for interaction = 0.05).In men, the risk of frailty levels linearly decreased with testosterone (adjusted OR for frailty = 2.9 (95%CI, 1.6–5.1) and 1.6 (95%CI, 1.0–2.5), for 1 SD decrease in TT and FT, respectively). TT and FT showed association with most of frailty criteria. No interaction was found with BMI.

**Conclusion:**

There is a relationship between circulating levels of FT and frailty in older women. This relation seems to be modulated by BMI. The relevance and the nature of the association of FT levels and frailty are sex-specific, suggesting that different biological mechanisms may be involved.

## Introduction

In the last decades the concept of frailty has emerged as one of the main conditions preceding the development of disability. Fried et al. have given the most widely used definition of frailty [1]: “frailty is an age-associated syndrome characterized by a reduced functional reserve and impaired adaptive capacity”. This phenotype of frailty has shown a strong association with increased risks of disability and other adverse outcomes: institutionalization, hospitalization, falls, and mortality [2], [3], [4].

This characterization of frailty stems from a physiological framework where sarcopenia and muscle performance play a pivotal role [5], [6]. The contribution of different components to the development of frailty (hormones, inflammation, oxidative stress, mithochondrial DNA, etc) has been largely controversial since its description [7]. Among hormones, low levels of endogenous testosterone, largely linked to the amount and quality of muscle mass, is one of the potential mechanisms involved in the development of frailty [8], [9]. Although the age-associated decline in testosterone occurs both in men and women [8], [10], this decline does not arise to the same extent in both sexes, suggesting a possible differential impact on frailty according to sex. To date, a consistent number of studies have reported an association between low testosterone levels and frailty in men [11], [12], [13]. However, although women represent around 2/3 of individuals with frailty, little is known on the impact of low testosterone on frailty or its components in women. To our knowledge, only two recent cross-sectional studies have examined this issue in post-menopausal women; while Cappola et al. [14] did not find free testosterone (FT) to be related to frailty, Wu et al. [12] reported an inverse association of TT (total testosterone) and FT with frailty in a small sample of Asian individuals.

In this context, using data from a population-based cohort of Spanish elders, we aimed to evaluate the sex-specific association between testosterone (total and free) and frailty.

## Methods

### Ethic statements

All study participants gave a signed informed consent. If a participant was not able to consent, his caregiver (member of his family or legal tutor) consented on his behalf. The study protocol was approved by the Clinical Research Ethics Committee of the Complejo Hospitalario de Toledo, Spain.

### Study population

**Figure 1:**
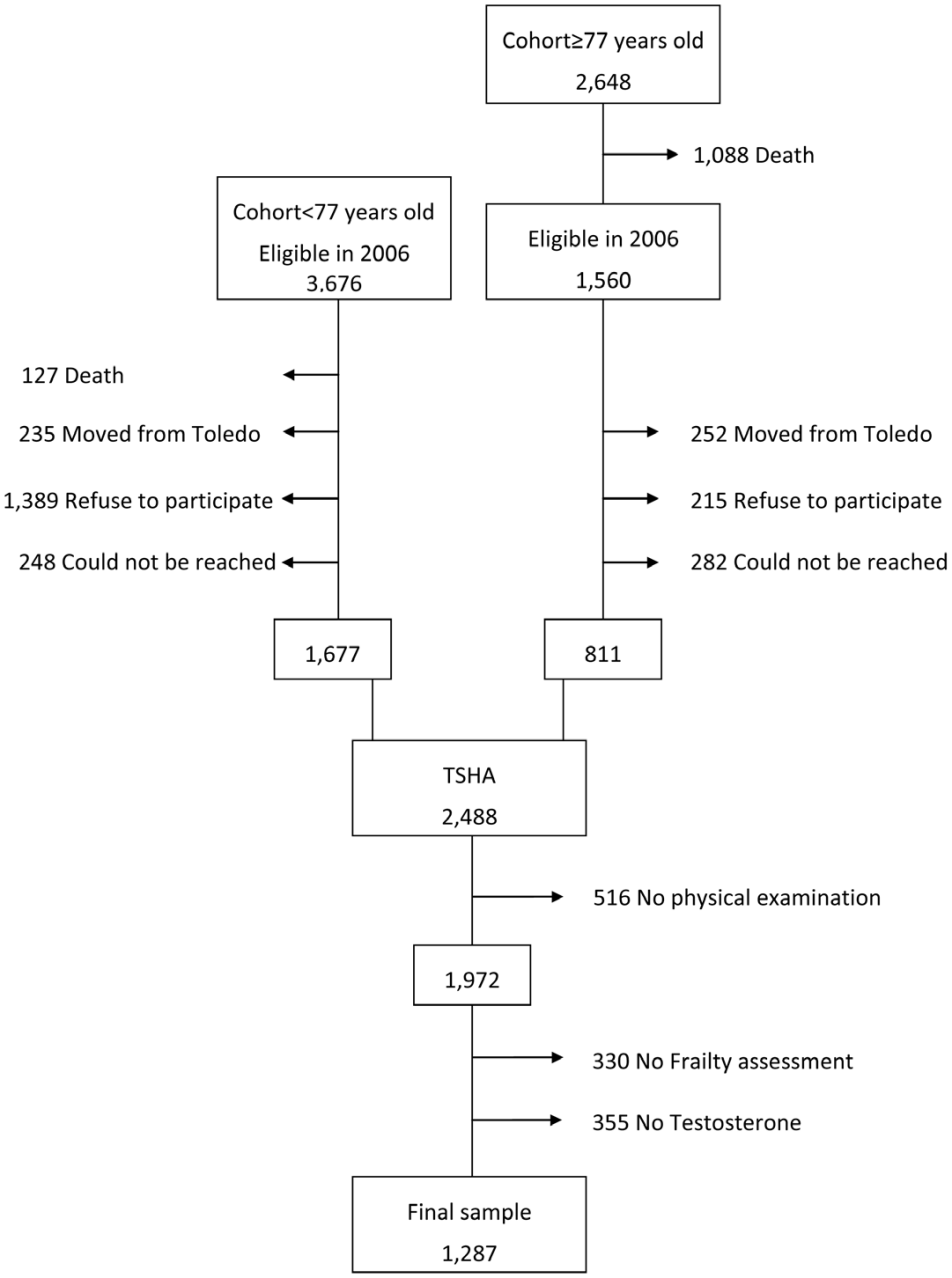
Flow Chart of the Study Sample Selection.

This analysis is based on the baseline data of the Toledo Study for Healthy Aging (TSHA), a Spanish longitudinal population-based cohort aimed at studying the determinants of frailty in older adults. The study methods have been reported elsewhere [15]. Briefly, the TSHA comprises two cohorts population. The first cohort is formed by the survivors of the “old cohort” called Toledo Study (a population-based cohort initiated in 1994); a population of men and women aged ≥77 years old in 2006 [16]. The second cohort is formed by individuals aged between 65 and 76 years-old specifically recruited for this study in 2006 (Figure 1). In both studies, subjects were selected by a two-stage random sampling from the Municipal Census of the province of Toledo. Of the previously existing Toledo Study 1,560 subjects were found to be eligible. Regarding the new cohort, 3,676 were initially selected. From these 5,236 individuals, and after exclusion of subjects who died, who moved from Toledo, refused to participate or could not be contacted, 2,488 subjects were included in the TSHA. This final sample showed similar demographic characteristics than the original sample. Of note, subjects selected from the “old cohort” received the same baseline assessment than subjects newly selected.

At baseline, data were collected by trained psychologists at the subjects' house. Questionnaires included socio-demographic characteristics, social support, limitations in activities of daily living (Katz index [17] and Lawton index [18]), health-related quality of life, physical activity, diet, alcohol use, and depressive symptoms and an extensive neuropsychology evaluation.

Study participants also underwent a physical exam performed by nurses specifically trained for the purpose of this study. Heart rate, blood pressure, anthropometric variables and the ankle-brachial index were measured. In addition, electrocardiogram, spirometry, and physical performance tests (upper and lower extremity strength, walk speed, balance and sit and stand from chair test) were performed. Information on comorbidity were self-reported, and include cardiovascular disease, hypertension, diabetes mellitus, high cholesterol, cancer, and dementia.

### Hormones measures

At baseline, all participants were invited to be drawn a sample of blood (45cc) while fasting. Samples were centrifuged and serum fraction taken to the laboratory within two hours, using containers at a temperature between 2 and 4°C, and then divided in aliquots and stored at −80°C. Serum concentration of TT and FT were measured in the laboratory of the Department of Biochemistry of the Hospital Universitario de Getafe (Madrid, Spain) using enzyme immunoassay techniques (ELISA) (DRG Testosterone, DRG Free Testosterone ELISA kits) in a DYNEX DS2 Automated ELISA System. Analytical sensitivity and intra- and interassay reagents ‘coefficient of variation were respectively: TT ELISA: 0.083 ng/ml; 3.34–4.16% and 4.73–9.94%; FT ELISA: 0.06 pg/ml; <10% and <10%. Values of TT and FT lower than the sensitivity threshold were given the value of this threshold in statistical analysis.

### Frailty measure

Frailty was defined, according to Fried's et al. approach [1], as the presence of three or more of the following components: slowness, weakness, weight loss, exhaustion, and low physical activity. Otherwise, subjects were classified as non-frail if no component were present, and as pre-frail if they had one or two of them. The components' definition has been described elsewhere [15]. Briefly, weight loss was defined as an unintentional loss of at least 4.5 kg during the last year, slowness was defined using the three-meter walking speed test, adjusted for sex and height according to the standards of the Short Physical Performance Battery [19]. To assess weakness, strength was measured with a Jaymar hydraulic dynamometer, according to the standards of the Hispanic EPESE [20]. Exhaustion was assessed using two questions (“I felt that anything I did was a big effort” and “I felt that I could not keep on doing things” at least 3 to 4 days a week”) of the Center for Epidemiological Studies Depression Scale [21]. Finally, low physical activity was defined as the worse quintile in the PASE scores [22].

Among the 1,972 subjects with a physical examination, 330 could not perform the physical performance test battery due to poor health and were excluded (Figure 1). As fully described in Garcia-Garcia et al. [15], the 1,972 subjects with physical examination were not different from the total TSHA cohort in terms of socio-demographic characteristics and comorbidities.

### Statistical analysis

Differences in testosterone levels according to subject's characteristics were assessed using Student t-test or ANOVA test. In case of non-normality, TT and FT variables were log-transformed.

Levels of TT and FT according to frailty status are expressed as age-adjusted geometric means (GM) and inter quartile range (IQR). Main study associations were summarized with odds-ratios (OR) of pre-frailty and frailty per 1 SD decrease in testosterone. Age and multivariate-adjusted OR and 95% confidence intervals (CI) were estimated from polytomous logistic regression. Adjustment included age, body mass index (BMI), education, hypertension, hypercholesterolemia, diabetes, and history of myocardial infarction or stroke. Because of the potential role of adipose tissue in the pathophysiological pathways leading to frailty, we seek for interaction of TT and FT with BMI on frailty. Finally, we display age-adjusted GM, IQR values of TT and FT according to each component of frailty and to the number of components. The association between each component of frailty and, TT and FT was assessed using age-adjusted logistic regression. ANOVA was used to compare the GM of TT and FT according to the number of components.

To examine for departure from linearity, analyses were run according to quartiles of hormones. Standard tests for linear trend and deviation from linearity were performed. We also tested quadratic terms for TT and FT to evaluate a possible U- or J-shaped relationship with frailty.

Analyses were conducted in each sex separately. Statistical significance was set at 2-sided p≤0.05. All analyses were performed using SAS statistical software version 9.2.

## Results

### Study population

From the 1,642 subjects where frailty was determined, 1,287 also had a measure of testosterone (552 were men and 735 women). Subjects without blood sample were not different from the others in terms of age, sex, and comorbidities [15]. The mean age of our study sample was 74.4. and 47.5% of the subjects had at least two cardiovascular risk factors. In addition, 9.1% had reported a previous myocardial infarction or stroke.

### TT and FT according to socio-demographic and medical variables

The median TT and FT values were 4.31 ng/ml and 6.05 pg/ml respectively in men, and 0.43 ng/ml and 0.38 pg/ml, respectively in women. The range and distribution of the data were different by sex (table 1).

**Table 1:** Means (standard deviation) of Total and Free Testosterone according to Subject's Sociodemographic and Medical Characteristics, by Sex.

		Men							Women						
		TT, ng/ml				FT, pg/ml			TT, ng/ml				FT, pg/ml		
		n	m	SD	p-value*	m	SD	p-value*	n	m	SD	p-value*	m	SD	p-value*
**Quartiles**	1	138	2.28	1.07		2.30	1.28		183	0.16	0.05		0.07	0.02	
**Testosterone**	2	138	3.89	0.27		5.08	0.65		184	0.33	0.05		0.23	0.08	
	3	138	5.01	0.42		7.52	0.83		184	0.53	0.07		0.60	0.16	
	4	138	7.26	1.45		14.9	7.83		183	1.14	1.06		2.43	2.22	
**Age**	<75	293	4.73	2.17	***0.029***	7.84	6.62	***0.005***	391	0.57	0.78	*0.148*	0.91	1.65	*0.215*
	[75–80[	172	4.52	1.78		6.73	5.00		225	0.53	0.39		0.78	1.25	
	≥80	87	4.39	2.06		7.52	6.49		118	0.47	0.57		0.69	1.11	
**BMI in class**	<25	94	4.87	2.46	***0.009***	8.09	7.33	*0.948*	98	0.54	0.69	*0.944*	0.76	1.53	*0.173*
	[25–30[	276	4.83	1.99		7.17	4.78		280	0.56	0.71		0.83	1.47	
	≥30	181	4.15	1.79		7.50	7.25		349	0.53	0.59		0.87	1.44	
**Educational level**	No formal schooling	374	4.56	2.05	*0.696*	7.67	6.69	*0.835*	485	0.52	0.48	*0.376*	0.84	1.41	*0.881*
	Uncompleted school	77	4.75	2.20		7.31	5.25		148	0.55	0.86		0.80	1.54	
	Primary or secondary school	100	4.68	1.88		6.71	4.45		99	0.63	0.95		0.84	1.57	
**Hypertension**	No	296	4.71	2.13	*0.238*	7.55	6.68	*0.581*	313	0.57	0.73	*0.468*	0.92	1.77	*0.92*
	Yes	249	4.50	1.95		7.32	5.53		415	0.52	0.58		0.78	1.18	
**Hypercholesterolemia**	No	371	4.79	2.10	***0.005***	7.95	6.96	***0.039***	395	0.60	0.82	***0.049***	0.91	1.50	*0.113*
	Yes	164	4.25	1.87		6.42	3.90		326	0.47	0.36		0.75	1.43	
**Diabetes**	No	434	4.69	2.08	*0.069*	7.56	6.51	*0.844*	596	0.55	0.71	*0.654*	0.79	1.42	*0.216*
	Yes	109	4.29	1.89		7.08	4.69		131	0.48	0.26		1.04	1.64	
**Myocardial infarction**	No	495	4.70	2.05	***0.001***	7.59	6.25	*0.061*	704	0.54	0.65	*0.658*	0.84	1.48	*0.665*
	Yes	54	3.75	1.62		6.15	5.16		27	0.60	0.64		0.69	0.98	
**Stroke**	No	527	4.63	2.03	*0.301*	7.43	6.06	*0.938*	708	0.54	0.64	*0.084*	0.85	1.47	*0.105*
	Yes	23	4.19	1.91		8.18	8.07		22	0.54	1.02		0.63	1.20	
**Dependance to BADL**	No	487	4.65	2.06	*0.149*	7.49	6.30	*0.684*	579	0.54	0.65	*0.173*	0.83	1.51	*0.328*
	Yes	60	4.25	1.78		6.89	4.80		147	0.50	0.53		0.84	1.19	
**Dependance to IADL**	No	100	4.78	1.77	*0.270*	7.90	5.01	*0.199*	401	0.48	0.29	*0.956*	0.74	1.09	*0.452*
	Yes	385	4.53	2.04		7.22	6.27		314	0.58	0.83		0.93	1.77	

*p-value are calculated from Student t-test or from ANOVA when the covariate had more than 2 categories, Significant differences are highlighted in bold.

Table 1 also displays the mean levels of TT and FT according to socio-demographic and medical characteristics in men and women separately. In men, TT decreased with age, obesity, hypercholesterolemia, and history of myocardial infarction. FT decreased with age and hypercholesterolemia. In women, TT and FT decreased with age but statistical significance was not reached (p = 0.148 and p = 0.215, respectively). Low levels of TT were associated with hypercholesterolemia (p = 0.049). FT was significantly associated with none of the characteristics studied but lower levels of FT were observed in subjects with a BMI<25 kg/m^2^ (p = 0.173) or with a history of stroke (p = 0.105).

### Association of TT and FT with frailty

Overall, 42.1% of the subjects were pre-frail and 8.3% frail. Although sex differences did not reach statistical significance (p = 0.114), we noted 6.5% of frail subjects in men versus 9.7% in women (table 2).

**Table 2:** Age-adjusted Geometric Means (GM)*, Inter Quartile Range (IQR) of Total and Free Testosterone Concentration according to Frailty Status, by Sex.

	Men			Women		
		TT, ng/ml	FT, pg/ml		TT, ng/ml	FT, pg/ml
	n (%)	GM, IQR	GM, IQR	n (%)	GM, IQR	GM, IQR
**Non frail**	275 (49.8)	4.43 (3.56–5.98)	5.92 (4.43–9.48)	363 (49.5)	0.41 (0.23–0.68)	0.37 (0.13–0.93)
**Prefrail**	241 (43.7)	3.70 (3.35–5.69)	5.00 (3.63–9.20)	300 (40.9)	0.40 (0.25–0.67)	0.37 (0.12–0.94)
**Frail**	36 ( 6.5)	2.45 (1.82–4.71)	2.80 (1.97–7.61)	71 ( 9.7)	0.36 (0.16–0.56)	0.27 (0.06–0.88)
p-value		<0.0001	0.003		0.474	0.196

*Age-adjusted GM were calculated using linear regression.

In men, TT decreased across frailty severity (table 2). Compared with non-frail, for 1 SD decrease in TT, the age-adjusted OR of being pre-frail was 1.35 times higher (95%CI, 1.08–1.70) and the OR of being frail was 1.79 times higher (95% CI, 1.31–2.44) (table 3). Results did not change significantly after adjustment for all socio-demographic characteristics and comorbidities. Frailty was also inversely associated with FT (age-adjusted OR = 1.59, 95% CI 1.18–2.15). The OR of being frail linearly decreased across quartiles of TT and FT (p for linear trend 0.005 and 0.037, respectively). No significant interactions was observed between TT, FT and BMI on frailty (p = 0.602 and 0.193, respectively).

**Table 3:** Relationship between Frailty Status and Total and Free Testosterone Concentration, in Men.

	Pre-frail (n = 241)			Frail (n = 36)		
	n (%)	OR (95%CI)	p-value	n (%)	OR (95%CI)	p-value
***TT, ng/ml, OR for 1 SD decrease***						
*Age adjusted*		1.35 (1.08–1.70)	**0.008**		1.79 (1.31–2.44)	**0.0002**
*Mutivariate adjusted* §		1.27 (1.00–1.62)	**0.042**		1.85 (1.31–2.62)	**0.0004**
***OR for quartiles*** §						
Q1	63 (45.7)	1.32 (0.77–2.25)	0.320	18 (14.5)	3.01 (0.98–9.29)	0.055
Q2	63 (45.7)	1.34 (0.79–2.27)	0.272	8 (5.5)	2.15 (0.63–7.37)	0.221
Q3	58 (42.0)	1.07 (0.64–1.81)	0.795	3 (2.2)	0.25 (0.04–1.45)	0.121
Q4	57 (41.3)	1.00		7 (4.8)	1.00	
p for trend			0.225			**0.005**
***FT, pg/ml, OR for 1 SD decrease***						
*Age adjusted*		1.17 (0.96–1.43)	0.108		1.59 (1.18–2.15)	**0.002**
*Mutivariate adjusted*†		1.14 (0.93–1.41)	0.191		1.67 (1.22–2.30)	**0.001**
***OR for quartiles*** §						
Q1	67 (48.6)	1.32 (0.78–2.23)	0.307	15 (11.3)	5.66 (1.41–22.8)	0.015
Q2	57 (41.3)	0.85 (0.50–1.41)	0.521	6 ( 4.4)	1.68 (0.37–7.70)	0.505
Q3	56 (40.6)	0.93 (0.55–1.56)	0.785	11 ( 7.8)	3.32 (0.80–13.9)	0.100
Q4	61 (44.2)	1.00		4 ( 2.9)	1.00	
p for trend			0.427			**0.037**

Results from Polytomous Regression Analyses.

§ *Adjusted for age, body mass index, educational level, hypertension, hypercholesterolemia, diabetes, history of myocardial infarction and stroke.*

*Significant differences are highlighted in bold.*

**Figure 2:**
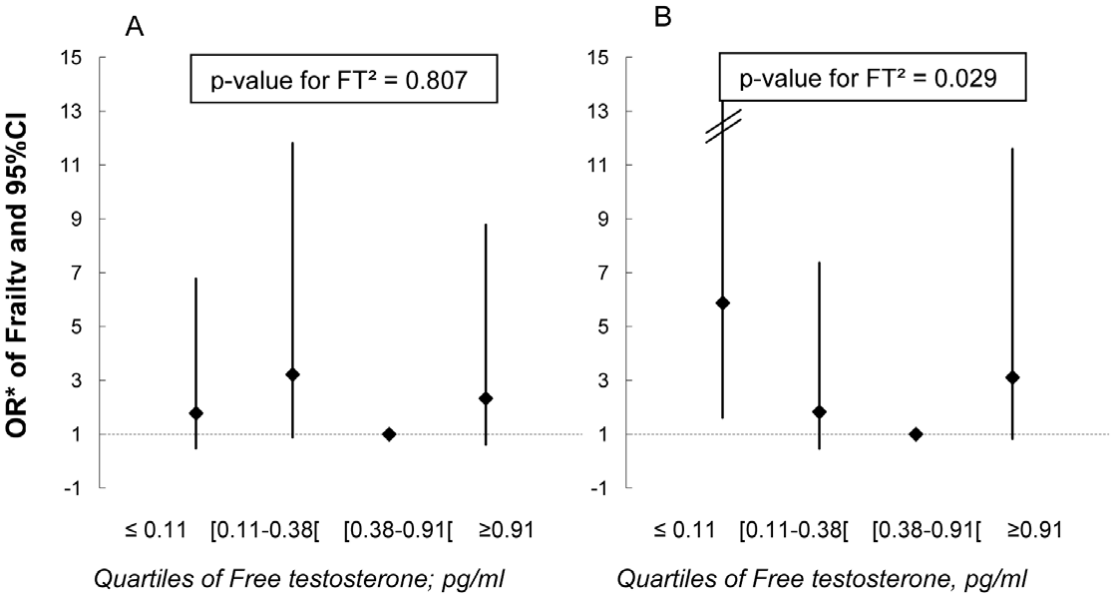
Multivariate Odds Ratios and 95% Confidence Interval of Frailty Associated with Quartiles of Free Testosterone, by Obesity, in Women. Part A corresponds to women with BMI<30 kg/m^2^. Part B corresponds to women with BMI≥30 kg/m^2^. ORs are adjusted for age, body mass index, educational level, hypertension, hypercholesterolemia, diabetes, history of myocardial infarction and stroke. Age-adjusted p-value for interaction between BMI (≥30 kg/m^2^) and FT^2^ = 0.050.

In women, FT was lower in frail subjects compared with non-frail or pre-frail but the association did not reach statistical significance (p = 0.196, table 2). When FT was classified into quartiles, deviation from linearity was observed (p = 0.009) and results suggested a U-shaped relationship between FT and frailty (p for FT^2^ = 0.029, table 4). Compared with non-frail, the risk of being frail was 2.69 times higher (95%CI 1.07–6.78) in women with the highest FT values and more than 3 times higher (95%CI 1.38–8.24) in women with the lowest FT values. No association was found between TT and frailty in women. Finally, we found evidence of an interaction between FT and obesity on frailty (Figure 2). The U-shaped relationship between FT and frailty was confined to women with obesity (p for interaction = 0.050).

**Table 4:** Relationship between Frailty Status and Total and Free Testosterone Concentration, in Women.

	Pre-frail (n = 300)			Frail (n = 71)		
	n (%)	OR* (95%CI)	p-value	n (%)	OR* (95%CI)	p-value
***TT, ng/ml, OR for 1 SD decrease***						
*Age adjusted*		1.02 (0.50–1.19)	0.772		1.15 (0.88–1.50)	0.297
*Mutivariate adjusted* §		1.00 (0.85–1.17)	0.968		1.13 (0.85–1.50)	0.378
***OR for quartiles*** §						
Q1	68 (37.2)	0.82 (0.51–1.31)	0.496	23 (12.3)	1.50 (0.66–3.44)	0.334
Q2	88 (47.8)	1.41 (0.40–2.22)	0.156	14 ( 7.8)	1.14 (0.46–2.83)	0.185
Q3	67 (36.4)	0.83 (0.52–1.32)	0.480	22 (11.9)	1.46 (0.63–3.38)	0.380
Q4	77 (42.1)	1.00		12 ( 6.6)	1.00	
p U-shaped			0.948			0.066
***FT, pg/ml, OR for 1 SD decrease***						
*Age adjusted*		1.00 (0.85–1.10)	0.925		1.27 (0.96–1.68)	0.091
*Mutivariate adjusted* §		1.00 (0.91–1.10)	0.905		1.22 (0.91–1.63)	0.180
***OR for quartiles*** §						
Q1	69 (37.7)	1.15 (0.72–1.84)	0.552	27 (15.1)	3.37 (1.38–8.24)	**0.008**
Q2	82 (44.8)	1.60 (1.01–2.54)	0.044	17 ( 9.6)	2.65 (1.05–6.78)	**0.042**
Q3	73 (39.7)	1.00		10 ( 5.2)	1.00	
Q4	75 (41.0)	1.27 (0.80–2.00)	0.308	17 ( 9.1)	2.69 (1.07–6.78)	**0.036**
p U-shaped			0.619			**0.029**

Results from Polytomous Regression Analyses.

§ *Adjusted for age, body mass index, educational level, hypertension, hypercholesterolemia, diabetes, history of myocardial infarction and stroke.*

*Significant differences are highlighted in bold.*

### Association of TT and FT with frailty components

In men, taking into account age, all components of frailty were associated with lower levels of TT and, low physical activity, weakness and weight loss with lower levels of FT (Table 5). In women, lower levels of TT and FT were observed in individuals presenting each component of frailty (except for weight loss where TT is slightly higher), but none of the age-adjusted associations was significant (table 6). Table 5 shows a smooth linear decline in TT and FT with the number of frailty components in men (age-adjusted p<0.0001 and p = 0.002, respectively) whereas only very low concentrations of FT were observed in women presenting more than 4 components (age-adjusted GM = 0.13 versus 0.37 in subjects with less than 4 components; age-adjusted p for ANOVA = 0.010, p for difference between women presenting more than 4 components and each one of the other categories was <0.001, table 6).

**Table 5:** Age-adjusted GM*, IQR value of total and free testosterone according to components and number of components of frailty, in Men.

	TT, ng/ml				TL. pg/ml			
	N	GM	IQR	p-value§	N	GM	IQR	p-value§
**Non-frail**	275	4.39	(3.56–5.98)	Ref	275	5.93	(4.48–9.48)	Ref
**Exhaution**	26	2.86	(2.88–4.05)	**<0.001**	26	4.31	(3.06–8.08)	0.090
**Low physical activity**	145	3.42	(3.03–5.41)	**0.002**	145	4.57	(3.63–8.41)	**0.029**
**Weakness**	102	2.89	(2.85–5.00)	**<0.0001**	102	3.71	(2.77–8.16)	**0.0005**
**Slowness**	126	3.42	(3.32–5.52)	**0.007**	126	4.81	(3.81–9.11)	0.079
**Weight loss**	39	3.16	(2.88–5.92)	**0.001**	39	3.67	(2.24–8.84)	**0.004**

Nb of components	N	GM	IQR	p-value#	N	GM	IQR	p-value#
**0**	275	4.39	(3.56–5.98)	**<0.0001**	275	5.93	(4.48–9.48)	**0.002**
**1**	161	4.06	(3.59–5.81)		161	5.37	(3.97–9.11)	
**2**	80	3.16	(3.12–5.25)		80	2.75	(3.25–9.20)	
**3**	29	2.56	(2.13–4.57)		29	2.51	(1.73–7.53)	
**4 or 5**	7	2.34	(0.79–5.81)		7	5.87	(2.88–8.49)	

*Age-adjusted GM were calculated using linear regression.

§ *Age-adjusted p-value were calculated using logistic regression.*

# *Age-adjusted p-value were calculated using ANOVA.*

*Significant differences are highlighted in bold.*

**Table 6:** Age-adjusted GM*, IQR value of total and free testosterone according to components and number of components of frailty, in Women.

	TT, ng/ml				TL. pg/ml			
	N	GM	IQR	p-value§	N	GM	IQR	p-value§
**Non-frail**	363	0.41	(0.23–0.68)	Ref	363	0.37	(0.13–0.98)	Ref
**Exhaution**	130	0.40	(0.29–0.62)	0.830	129	0.30	(0.08–0.86)	0.100
**Low physical activity**	82	0.41	(0.19–0.62)	0.964	82	0.36	(0.06–1.00)	0.941
**Weakness**	146	0.35	(0.22–0.58)	0.059	146	0.32	(0.09–0.88)	0.311
**Slowness**	178	0.36	(0.20–0.61)	0.076	176	0.32	(0.09–0.95)	0.187
**Weight loss**	84	0.43	(0.28–0.68)	0.523	84	0.35	(0.14–0.79)	0.740

Nb of components	N	GM	IQR	p-value#	N	GM	IQR	p-value#
**0**	363	0.41	(0.23–0.68)	0.470	363	0.37	(0.13–0.98)	**0.010**
**1**	218	0.39	(0.23–0.65)		219	0.36	(0.12–0.87)	
**2**	82	0.41	(0.28–0.71)		80	0.40	(0.13–1.11)	
**3**	50	0.39	(0.18–0.61)		50	0.37	(0.08–1.07)	
**4/5**	21	0.30	(0.16–0.53)		21	0.13	(0.06–0.22)	

*Age-adjusted GM were calculated using linear regression.

§ *Age-adjusted p-value were calculated using logistic regression.*

# *Age-adjusted p-value were calculated using ANOVA.*

*Significant differences are highlighted in bold.*

### Sensitivity analysis

We performed a sensitivity analysis excluding all men taking a treatment for prostate cancer or adenoma (n = 62). Although these men had slightly higher means of TT (5.0 (3.7) versus 4.6 (3.4), p-value = 0.172) and FT (9.8 (5.1) versus 7.1 (3.9), p = 0.039), univariate and multivariate analysis regarding the association of TT and FT with frailty were not modified (data not shown). No women were using hormonal therapy in our study.

## Discussion

In a large sample of Spanish community dwellers, we found, for the first time in a Caucasian population, a significant U-shaped relationship between circulating levels of FT and frailty in older women. In addition, we highlighted different patterns of association of FT with frailty and its components according to sex.

We found a negative association of TT and FT with frailty in men. This result confirms those from other observational studies. Indeed, several studies have found a cross-sectional association between low levels of bioavailable or FT and frailty or the severity of its components [11], [12], [23], [24], [25]. Regarding TT, results are less consistent across studies, some of them suggesting an association [12], [23], [25] and other not [11], [13], [24]. Furthermore, we found a strong association between each component of frailty and TT in men; lack of physical activity, slowness and weight loss were also associated with decreased FT. These results are consistent with other studies reporting association of TT and FT with muscle strength [11], [13], [23], [24], [25], walking speed [11], [24], and weight loss [23].

We also report a U-shaped relationship between FT and frailty in women. Higher and lower levels of FT were associated with an increased probability of being frail. This result is different from the one from Cappola et al. [14] which suggested a possible (but not significant) linear association between decreasing testosterone and frailty.

Interestingly, we found various differences in the relationship between testosterone and frailty according to sex. Firstly, we showed that the probability of frailty linearly increased with testosterone's decline in men, while this relationship was U-shaped in women. Secondly, analyses regarding the components of frailty revealed a strong association between each component and TT in men whereas, in women, no single component was associated neither with TT nor with FT. Thirdly, while FT levels progressively decrease with the number of frailty criteria in men, they sharply decreased in women with 4 or 5 criteria. Finally, obesity modified the effect of FT on frailty in women but not in men.

The implication of testosterone in body-mass regulation, muscle function and growth and, regulation of bone mineral density [25], [26], [27], [28] gives biological support to the relation between testosterone decline and frailty. Additionally, testosterone may be linked to weight loss through its effect on appetite [29]. However, the mechanisms underlying sex differences and, the association of higher levels of FT with frailty in women are not clear and need to be explored in other studies. One explanation for the disparity between sexes could be the implication of different biological mechanisms in the relation between testosterone and frailty. While testosterone plays a prominent role in frailty in old men, its role in women, although present, seems less relevant. Other hormonal axes and mediators may be of major importance in the relation between testosterone and frailty in women. This is supported by data from the Women's Health and Aging Study where a multiple hormonal burden was found to be more strongly associated with frailty than the type of hormonal deficiency [14]. Moreover, if testosterone plays a crucial role in men health, estrogens are the most important sex-hormones in women. Considering that the main source of estradiol in post-menopausal women comes from the conversion of testosterone by aromatase in adipose tissue, and that estrogen therapy and endogenous estrogen have been suggested to respectively have a positive effect on muscle strength [30], [31] and on bone mineral density [32], we can hypothesized that estradiol may play a substantial role in the relation between FT and frailty in women. Our results regarding the interaction between FT and obesity in women but not in men reinforce this hypothesis as they suggest a more important role of the estrogenic climate in women than men.

The strengths of our study mainly pertain to our large sample size of randomly selected community dwellers and to the well validated criteria used for the definition of frailty. In addition, although FT is considered the more biologically active hormone, nearly none of the previous studies have investigated the true amount of FT but used a calculated estimation. Even if the methods used to calculate free testosterone are recognized reliable [33], we cannot exclude that it could explain the difference between our study and others'. Nonetheless, the method used to measure testosterone also represents our main limitation. Indeed, levels of testosterone are very low in postmenopausal women, and thus the use of a highly sensitive assay may have been more appropriate. However, as all value of FT under the detection threshold were set to this threshold value, we are more likely to underestimate the true association between low levels of FT with frailty. In addition, when removing all women with a value of FT under the threshold of detection from the analyses (n = 132), the result remains similar (data not shown). Other important limitation is the observational and transversal characteristics of our study which do not allow us to conclude in terms of causality. Finally, although we adjusted our analyses for many confounding factors, we cannot exclude that the finding of a U-shaped relationship between FT and frailty may be the results of unmeasured confusion.

To conclude, our results show, for the first time, an implication of testosterone in the frailty syndrome in women, confirm the association of testosterone with frailty in men, and suggest a differential association of testosterone with frailty by sex. In light of the recent findings regarding the benefit of testosterone supplementation on the various component of frailty, our data, if confirmed, may have clinical implication. Indeed, most, if not all, clinical trials suggesting that maintaining testosterone in a normal range may contribute to prevent frailty have been performed in men with hypogonadism [28], [34], [35], [36], [37]. Here, we suggest that even without hypogonadism, men with low levels of testosterone are at increased risk of physical frailty and could thus benefit from testosterone therapy. In addition, postmenopausal women might also benefit from testosterone administration in the context of a wider hormonal care.
